# Development and field evaluation of a multiplex qPCR assay for environmental DNA detection of *Schistosoma mekongi* and its intermediate snail host *Neotricula aperta* in the Mekong River Basin

**DOI:** 10.1186/s40249-026-01466-1

**Published:** 2026-06-04

**Authors:** Jian He, Peter S. Andrus, Sengrloun Phonekeo, Kun Yin, Leshan Xiu, Shan Lv, Kun Yang, Somphou Sayasone, Xiao-Nong Zhou

**Affiliations:** 1 School of Global Health, Chinese Center for Tropical Diseases Research, Shanghai Jiao Tong University School of Medicine, Shanghai, China; 2 School of Public Health, Shanghai Jiao Tong University School of Medicine, Shanghai, China; 3 National Health Commission Key Laboratory of Parasitic Disease Control and Prevention, Jiangsu Provincial Key Laboratory on Parasite and Vector Control Technology, Jiangsu Provincial Medical Key Laboratory, Jiangsu Institute of Parasitic Diseases, Wuxi, Jiangsu China; 4 Academy of Pharmacy, Xi’an Jiaotong-Liverpool University, Suzhou, China; 5 Lao Tropical and Public Health Institute, Ministry of Health, Vientiane, Lao PDR; 6 National Institute of Parasitic Diseases at Chinese Center for Disease Control and Prevention (Chinese Center for Tropical Diseases Research); NHC Key Laboratory of Parasite and Vector Biology; WHO Collaborating Centre for Tropical Diseases; National Center for International Research on Tropical Diseases, Ministry of Science and Technology, Shanghai, 200025 China; 7 School of Public Health, Nanjing Medical University, Nanjing, China; 8 Hainan Center for Tropical Diseases Research (Hainan Subcenter of Chinese Center for Tropical Diseases Research), Haikou, China

**Keywords:** Environmental DNA, *Schistosoma mekongi*, *Neotricula aperta*, Multiplex qPCR, Environmental surveillance, Schistosomiasis elimination

## Abstract

**Background:**

*Schistosoma mekongi* remains an important cause of intestinal schistosomiasis in the lower Mekong River Basin. Sensitive environmental surveillance tools are needed to support transmission monitoring, particularly where conventional snail surveys are seasonally constrained. This study developed and evaluated a multiplex quantitative PCR (qPCR) assay for simultaneous detection of *S. mekongi* and its intermediate snail host, *Neotricula aperta*, in aquatic environmental DNA (eDNA) samples.

**Methods:**

Singleplex and multiplex qPCR assays targeting *S. mekongi* and *N. aperta* were compared to establish the optimal amplification system. Field validation was conducted in the Khong District, Champasak Province, Laos, during the dry season (May 2024) and wet season (August 2024) across 20 transmission sites. Method agreement between eDNA detection and malacological surveys was assessed using Cohen’s kappa coefficient, and associations between *N. aperta* density and physicochemical variables were evaluated using Spearman’s rank correlation.

**Results:**

The optimized multiplex qPCR assay targeting *N. aperta* (16S rRNA) and *S. mekongi* (18S rRNA) showed high sensitivity, with limits of detection of 5 and 6 copies/μl and limits of quantification of 40 and 46 copies/μl, respectively. In mesocosms, both targets were detected at an infected-snail density of 1 snail/L, with median Cq values of 35.23 for *N. aperta* and 26.54 for *S. mekongi*. During the dry-season surveys, 978 *N. aperta* were collected, with a mean density of 46.25 snails per site; wet-season surveys were not feasible. No schistosome infections were detected by crushing microscopy. Field eDNA analysis detected *N. aperta* but not *S. mekongi*, with *N. aperta* eDNA positivity being 90% in the dry season and 40% in the wet season. Agreement with malacological surveys was moderate (*κ* = 0.44; *p* = 0.01) and snail density was significantly associated with water temperature, pH, and dissolved oxygen.

**Conclusions:**

This multiplex eDNA assay sensitively detected *N. aperta* in field water samples and may help overcome seasonal limitations of malacological surveys. Although *S. mekongi* was detected under laboratory conditions, it was not detected in field samples, consistent with the absence of infected snails. Further validation in active-transmission sites is required before reliable field detection of *S. mekongi* eDNA can be assumed.

**Graphical Abstract:**

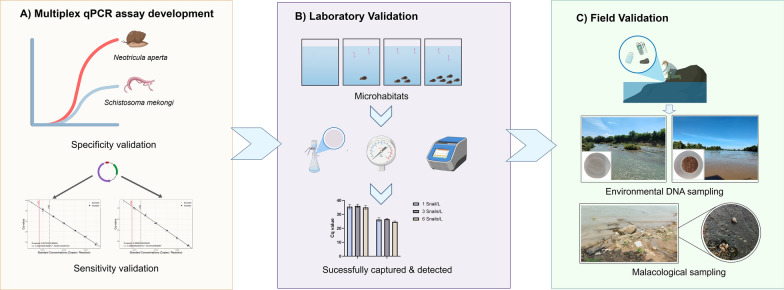

**Supplementary Information:**

The online version contains supplementary material available at 10.1186/s40249-026-01466-1.

## Background

Schistosomiasis remains a formidable waterborne parasitic disease of global public health significance [1], with an estimated 1,746,333 disability-adjusted life years (DALYs) attributed to the disease in 2021 [2]. In 2022, the *One Health: Approach for action against neglected tropical diseases (NTDs) 2021–2030* framework was released by the World Health Organization (WHO), emphasizing the need to improve understanding of human-animal-environmental transmission pathways of NTDs [3]. Among the six human *Schistosoma* species, *S. mekongi* is a significant zoonotic parasite distributed along a limited part of the Mekong River, primarily in Cambodia and the Lao People’s Democratic Republic [4]. In addition to humans, transmission is maintained by animal reservoirs, with dogs and pigs serving as the primary definitive hosts in endemic areas [5]. These zoonotic reservoirs, coupled with the intermediate snail host, *Neotricula aperta*, create complex transmission cycles that challenge elimination efforts. Despite achieving low endemicity in recent years, the region’s 2025 elimination goal requires sensitive and scalable tools to verify transmission interruption, a challenge compounded by the limitations of current surveillance methods [4].

Current environmental risk assessment for *S. mekongi* transmission primarily relies on morphological surveys of the intermediate snail host (*N. aperta*), supplemented by cercarial shedding assays to determine infection status and estimate transmission risk in aquatic habitats [5]. However, this approach presents several critical limitations. First, field sampling is inherently constrained by the small adult size of the snail (~ 3 mm shell length) and seasonally restricted accessibility (dry season only) [6, 7], resulting in an inability to monitor wet-season transmission dynamics when floodwaters disperse snail populations into tributaries. Second, the typically ultralow prevalence of infected snails (< 1% in most endemic foci) [8], combined with the need for specialized malacological expertise [9], severely compromises detection sensitivity. Third, current malacological surveys provide a fragmented view of the transmission landscape, as they often fail to account for zoonotic animal reservoirs, such as dogs and pigs, contributing to environmental egg contamination. These operational challenges render current methods inadequate for meeting elimination verification requirements [10]. Consequently, the development of a seasonally robust and highly sensitive environmental surveillance technology capable of supporting environmental assessment of transmission risk represents an urgent public health priority.

Environmental DNA (eDNA) refers to genetic material derived from all organisms present in an environmental sample [11], existing in both organismal and extra-organismal forms [12]. This technology provides an efficient, non-invasive and potentially standardized approach for assessing disease transmission risk at the human-environmental interface [13, 14]. However, standardized sampling protocols for schistosomiasis eDNA, specifically regarding optimal sampling volume, timing, and site selection, have yet to be established. Furthermore, the performance of eDNA assays is strongly influenced by seasonal hydrophysical variations, such as water temperature, pH, and flow dynamics, which affect eDNA degradation and transport across complex riverine landscapes [15]. Understanding these environmental drivers is critical for interpreting surveillance data, particularly in highly dynamic systems like the Mekong River. In the context of schistosomiasis, eDNA-based detection technology has emerged as a valuable surveillance tool in recent years [16]. Studies applying eDNA detection to *S. japonicum*, *S. haematobium*, and *S. mansoni*, along with their respective intermediate hosts, have been conducted across multiple endemic regions [17–21]. Despite these advances, eDNA methodologies have not yet been integrated into long-term national or international monitoring strategies, and they currently lack the formal guidelines required for routine public health use [22]. No comparable eDNA tools currently exist for *S. mekongi*, leaving schistosomiasis elimination efforts in the Mekong River Basin without this innovative molecular surveillance tool.

In this study, we report the development, laboratory validation and field evaluation of a multiplex quantitative PCR (qPCR) assay and water sampling strategy for detecting *S. mekongi* and *N. aperta* eDNA, incorporating a physicochemical baseline characterization to demonstrate the tool's seasonal robustness. By integrating controlled laboratory experiments with large-scale field sampling across transmission foci in Lao PDR, we demonstrate a novel, ecology-integrated approach to schistosomiasis surveillance. This methodology not only addresses immediate elimination challenges in the Mekong Basin but also supports the broader development of eDNA methodologies for monitoring schistosomiasis transmission in other endemic regions.

## Methods

### Development of the multiplex qPCR assay for *S. mekongi *and *N. aperta*

#### Primers and probe design and validation

Target regions for *S. mekongi* and *N. aperta* were selected from available reference sequences in GenBank. Candidate regions were screened to identify conserved target sites within each species while avoiding regions with high similarity to closely related taxa. Four candidate primer-probe sets were then designed for each target organism using Primer Express 3.0.1 (Applied Biosystems, Foster City, USA), resulting in eight candidate sets in total (Supplementary Table S1). Specific primers (18–24 nt, *T*_m_ 58–62 °C) and TaqMan-MGB probes (Minor Groove Binder modified, *T*_m_ 66–74 °C) were designed with 35–60% GC content. Amplicon lengths were restricted to 60–120 bp to maximize multiplex amplification efficiency. Primer and probe specificity was then evaluated in silico using the Primer-BLAST tool (National Center for Biotechnology Information, Bethesda, USA; available at https://www.ncbi.nlm.nih.gov/tools/primer-blast/) to assess potential cross-reactivity with closely related *Schistosoma* species, sympatric freshwater snails, and the human genome (*Homo sapiens*, Taxid: 9606). The selected primers and probes were synthesized by Sangon Biotech (Shanghai, China).

#### Genomic DNA extraction and multiplex qPCR detection

In addition to the field-collected snail species from *N. aperta* habitats (Supplementary Table S2), genomic DNA (gDNA) was also extracted from experimentally infected *N. aperta* (kindly provided by Mahidol University, Thailand) to serve as a positive template for assay optimization. All extractions were performed using the DNeasy^®^ Blood & Tissue Kit (QIAGEN, Hilden, Germany) following the manufacturer’s protocol. DNA concentration and purity were assessed using a K5600C microspectrophotometer (Kaiao Technology, Beijing, China). All qPCR assays were performed on a QuantStudio 7 Pro System (Applied Biosystems, Foster City, USA). Each 20 μl reaction contained 10 μl of 2 × AceQ qPCR Probe Master Mix (Vazyme Biotech, Nanjing, China), 1 μl of template DNA, 0.4 μl of each primer (10 μmol/L stock), and 0.2 μl of each probe (10 μmol/L stock). For the multiplex assay, separate *Taq*Man probes were used for each target to ensure independent detection. To ensure independent multiplex detection, the *N. aperta* and *S. mekongi* probes were labeled with FAM and VIC at the 5' end, respectively. Additionally, both probes incorporated a Minor Groove Binder (MGB) modification at the 3' end to elevate the *T*_m_ and maximize target discrimination. The final volume was adjusted to 20 μl with nuclease-free water.

To optimize the multiplex system, the initial performance of the designed primer-probe sets was evaluated using singleplex qPCR reactions. Subsequently, a combination was considered optimal if the Cq shift (ΔCq) between singleplex and multiplex assays (using the same *N. aperta* gDNA template) was < 2 cycles, indicating acceptable interference levels [23]. The multiplex assay specificity was also subsequently validated using genomic DNA from other *Schistosoma* species (*S. haematobium*, *S. japonicum*, and *S. mansoni*; kindly provided by Jiangsu Institute of Parasitic Diseases, China) and other field-collected snail species (Supplementary Table S2).

Thermal cycling conditions were performed according to the standardized two-step protocol recommended for TaqMan assays: initial polymerase activation at 95 °C for 5 min, followed by 50 cycles of denaturation at 95 °C for 10 s and combined annealing/extension at 60 °C for 30 s, with a final hold at 4 °C.

#### Standard curve construction and sensitivity analysis

Target sequences of *S. mekongi* and *N. aperta* were synthesized and cloned into pUC57 vectors by Sangon Biotech (Shanghai, China). The purified plasmid DNA was quantified using a K5600C microspectrophotometer (Kaiao Technology, Beijing, China). The copy number of the plasmid stock was calculated based on the molecular weight of the total nucleotide sequence.

$$Number of copies =\frac{(Amount in ng \times 6.022\times {10}^{23})}{(Length in bp \times {10}^{9}\times 660)}$$

Serial ten-fold dilutions of recombinant plasmid DNA containing target sequences (1–1 × 10⁶ copies/μl) were prepared to generate standard curves. For positive controls, infected *N. aperta* gDNA was used. Nuclease-free water was used as the negative control. The limits of detection (LoD) and quantification (LoQ) were calculated following Klymus et al. [24], based on triplicate measurements across three independently prepared standard curves .

### Laboratory validation of eDNA sampling methodology

To validate the eDNA collection protocol, controlled mesocosm experiments were conducted to simulate *N. aperta* microhabitats. Laboratory-reared infected snails, originally obtained from Mahidol University (Thailand), were pre-screened via the cercarial shedding method to confirm active *S. mekongi* infection. These confirmed shedding snails were maintained in density-controlled treatments (0, 1, 3, and 6 snails per 1 L container; *n* = 12 total) under simulated endemic environmental conditions (29 ± 1 °C; natural light). After a 24-h incubation period, 200 ml water samples were collected from each mesocosm [25]. Samples were vacuum-filtered through 0.7 μm glass fiber filters (Whatman GF/F 1825-047) using sterile filtration apparatus [17, 19]. All filters underwent DNA extraction within 24 h. For water samples, a modified extraction protocol was used: each filter was finely minced with sterile scissors and processed with increased ATL buffer (720 μl) and Proteinase K (80 μl) volumes [26]. Filters were aseptically cut into small fragments (approximately 1–2 mm^2^) using sterile scissors before being submerged in the lysis buffer to maximize DNA recovery. To prevent cross-contamination, eDNA extraction from water samples and DNA extraction from tissue were performed in separate laboratories. Water samples were collected/prepared as three independent biological replicates. Each water sample was also analyzed in three technical replicates by multiplex qPCR.

### Field sampling

#### Sampling sites

Field surveys were conducted in May (dry season) and August (wet season) 2024 across four Mekong River islands (Done Som, Done Xangphai, Done Long, and Done Kadem) in Champasak Province, southern Laos, which were specifically selected as well-documented, highly endemic transmission foci for *S. mekongi* and its intermediate snail host to provide an ideal real-world setting for validating the eDNA tool. A total of 20 sampling sites were established, with five sites distributed across each of the four islands.

#### Measurement of environmental parameters

At each site, water quality parameters, including pH, temperature, dissolved oxygen, and electrical conductivity, were measured using a calibrated AZ86031 multiparameter probe (AZ Instrument Corp., Taiwan, China). This data was recorded to provide an environmental baseline for assessing eDNA dynamics under contrasting hydrological conditions (dry vs. wet seasons), which is essential for understanding how factors like seasonal dilution and water flow influence eDNA persistence and detection sensitivity [27].

#### eDNA field collection and sample processing

For eDNA sampling, triplicate 1 L water samples were collected between 10:00 and 15:00 from human-contact zones, maintaining at least 10 m spacing between collection points [28, 29]. Using sterile procedures, subsurface water was collected in pre-cleaned polypropylene containers. To prevent eDNA degradation, the water containers were immediately stored in dark coolers with ice packs and transported to our local field laboratory for subsequent processing.

Samples were vacuum-filtered through 0.7 μm GF/F filters. While our target filtration volume was exactly 1 L per biological replicate, the elevated water turbidity of the Mekong River (especially during the rainy season) occasionally caused single filters to clog. In such instances, a second filter was routinely used to complete the filtration of that specific 1 L sample. Following filtration, all filters were stored at –20 °C in monitored freezers and extracted within 7 days.

eDNA extraction and analysis were conducted following the protocol described in Sect. "Laboratory Validation of eDNA Sampling Methodology". For 1 L samples that required multiple filters, a serial elution approach was employed during the final extraction step. This ensured that the resulting DNA eluate accurately pooled and represented the total eDNA captured from the original 1 L water volume.

#### Malacological sampling protocol

Following water collection, five flat rocks (covering approximately 1 m^2^ in total) were retrieved from the shore at each site. All snails attached to the rocks were washed into a tray and counted [30]. Collected snails were morphologically identified, followed by examination for *Schistosoma* infection using the crush-slide microscopy method to maximize detection sensitivity [31].

### Data analysis

Data was managed using Microsoft Excel 365 (Microsoft Corp., Redmond, USA). The geographic coordinates of all field survey sites, including longitude and latitude, were recorded using a handheld GPS receiver. Statistical analyses, including Cohen’s kappa (κ) for method agreement and Spearman’s rank correlation for environmental associations, were conducted using SPSS version 26.0 (IBM Corp., Armonk, USA) and GraphPad Prism version 9.0 (GraphPad Software Inc., San Diego, USA).

## Results

### Primer and probe specificity

**Fig. 1:**
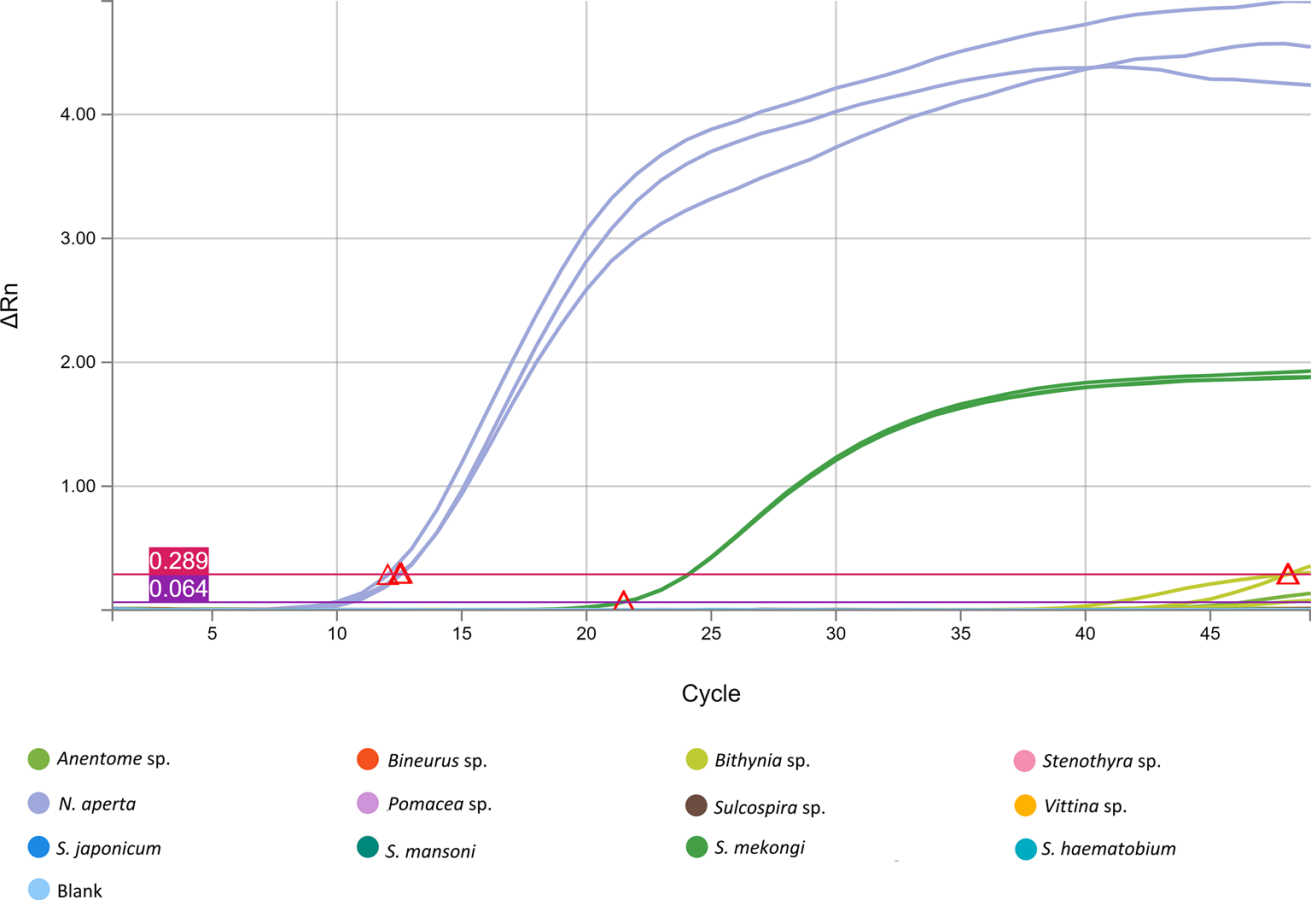
Primers and probes for detection of *Schistosoma mekongi* and *Neotricula aperta* and their cross-species amplification

Following comprehensive pairwise comparisons of the candidate primer-probe sets (Supplementary Table S3), the optimal combination for multiplex qPCR was identified. The best primer-probe sets for multiplex qPCR targeted the 18S ribosomal RNA gene of *S. mekongi* (GenBank: U89871.1) and the 16S rRNA gene of *N. aperta* (GenBank: MF663277.1; Table 1). In silico analysis using NCBI BLAST demonstrated 100% sequence homology with target regions of *S. mekongi* and *N. aperta*, while showing ≥ 3 nucleotide mismatches with non-target species (Supplementary Fig S1). In silico BLAST analyses against the *Homo sapiens* genome confirmed the assay’s high specificity. No significant homology was identified for *S. mekongi*, whereas remote similarity was noted for *N. aperta* (Accession: EU863790.1), non-specific amplification is precluded by a critical 3' terminal mismatch and an incompatible product size (870 bp). Thus, the assay is robust against interference from host-derived DNA. Experimental validation confirmed the high analytical specificity of the multiplex primer-probe sets, which amplified the target template (infected *N. aperta* gDNA) with mean Cq values of 12.46 for *N. aperta* and 21.50 for *S. mekongi*. Importantly, the assay demonstrated no meaningful cross-reactivity with other human-infecting *Schistosoma* species or with sympatric Mekong mollusks. Although a remarkably late amplification signal (Cq = 48.30) was observed for one *Bithynia* sp. sample, this value falls well beyond the reliable detection threshold and is considered non-specific background noise, further confirming the assay’s specificity (Fig. 1).

**Table 1:** Primer-probe sets designed for the multiplex qPCR assay of *Neotricula aperta* and *Schistosoma mekongi*

Species	Sequences	Reporter	Fragment length	Target gene
*N. aperta*	F: AAACGGCCGCGGTACTCT R: CCGTTCATACAAGCCCTCAATT P: ACCGTGCAAAGGTAG	FAM	74	16S rRNA
*S. mekongi*	F: CATGCACCTGGCCTTGTG R: TCGCTGCAGCCTAGGATATTTAC P: TGCATGTACGCTGGCT	VIC	68	18S rRNA

*F* Forward primer, *R* Reverse primer, *P* Probe

### Primer and probe sensitivity

**Fig. 2:**
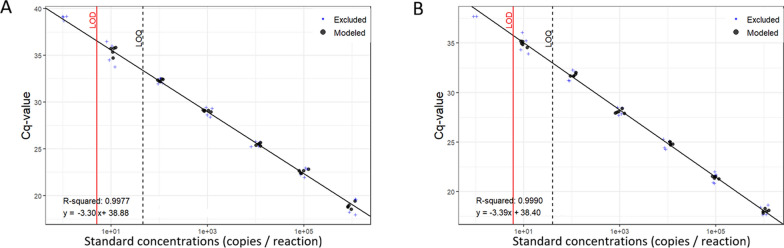
Standard curves showing the limits of detection (LoD) and quantification (LoQ) for *Neotricula aperta* and *Schistosoma mekongi* DNA assays. **A** *N. aperta* standard curve (data calculated following Klymus et al. [24]). Black circles indicate data points included in linear regression; blue plus signs ( +) denote excluded points. **B** *S. mekongi* standard curve

The quantitative reliability of the multiplex qPCR was confirmed by the standard curves, which exhibited a strong linear correlation between Cq values and log-copy numbers (> 0.99 for both targets) over a 6-log dynamic range (Fig. 2). The amplification efficiencies of *N. aperta* and *S. mekongi* were 101.03% and 97.23%, respectively. The mean Cq values ranged from 18.94 ± 0.55 (106 copies/μl) to 35.75 ± 0.81 (101 copies/μl) fo*r N. aperta*, and from 18.09 ± 0.32 (106 copies/μl) to 34.93 ± 0.61 (101 copies/μl) for *S. mekongi.* At the lowest dilution of 1 copy/μl, inconsistent amplification was observed (Cq > 37.70 or no amplification). Detailed raw Cq data for each replicate are provided in Supplementary Table S4.

The LoD and LoQ were determined using a probit regression analysis with an automatic model selection based on minimal residual standard error. For *N. aperta*, the Weibull 2.2-parameter model yielded an LoD of 5 copies/μl, while a fifth-order polynomial model established an LoQ of 40 copies/μl. Corresponding values for *S. mekongi* were 6 copies/μl (Aranda-Ordaz 2-parameter model) and 46 copies/μl (fifth-order polynomial), respectively, with a 35% coefficient of variation (CV) threshold for LoQ determination (Fig. 2). When tested under controlled mesocosm conditions, the 0.7 μm glass fiber filters successfully captured genetic material from 200 ml water samples containing a single infected *N. aperta*, yielding consistent dual-positive signals for both *S. mekongi* and *N. aperta* via multiplex qPCR detection (Supplementary Fig S2).

### Morphological survey results

A two-phase field investigation was conducted in the Khong District, Champasak Province, southern Laos. During the dry season (May 2024), 60 water samples were successfully collected for eDNA detection. Malacological surveys at 20 selected sites (five per island) detected *N. aperta* at 90% of locations (18/20). A total of 978 *N. aperta* individuals and 685 other co-occurring mollusks were collected and identified morphologically. The mean density of *N. aperta* was 46.25 (± 39.77) individuals per site. When testing for infection, no *Schistosoma*-infected *N. aperta* were detected by microscopy.

During the following wet season (August 2024), however, malacological surveys were technically unfeasible due to extreme monsoon conditions, including significantly elevated water levels and rapid flow rates, which prohibited safe and effective snail sampling.

### eDNA spatiotemporal dynamics

Due to the logistical convenience of the eDNA sampling protocol, we successfully collected 60 water samples (three biological replicates per site) during both the dry (May 2024) and wet (August 2024) seasons. During dry-season sampling (May 2024), *N. aperta* eDNA was detected at 90% of sites (18/20; Table 2). The Cohen's Kappa coefficient (*κ* = 0.44,* P* < 0.05) indicated moderate agreement between the eDNA and malacological results, despite a high observed agreement of 90%. The discrepancy was mainly due to the low prevalence of negative cases (2/20), which artificially inflated the expected chance agreement. During the wet season (August 2024), *N. aperta* eDNA was detected at 40% of sites (8/20), with significantly higher mean Cq values (37.8 ± 1.2; *t-*test *P* < 0.05 vs. dry season). Across both seasons, no *S. mekongi* eDNA was detected, yielding 100% negative concordance across all 120 water samples analyzed.

**Table 2:** Survey metrics of *Neotricula aperta* detection using malacological and eDNA methods across four islands in Lao PDR

Islands	Village	GPS location	Number of *N. aperta* (Morphological)		Positive water samples (Biological replicates, *n* = 3)		Positive qPCR reactions (Technical replicates, *n* = 9)	
			May	Aug	May	Aug	May	Aug
Done Som	Hangsom	13.9874N 105.9083E	12	–	1/3	2/3	3/9	5/9
	Hangsom	13.9973N 105.9101E	6	–	2/3	0/3	6/9	0/9
	Somvenok	14.0138N 105.9027E	13	–	2/3	0/3	6/9	0/9
	Thamarkaep	14.0208N 105.8991E	4	–	1/3	0/3	3/9	0/9
	Thamarkaep	14.0268N 105.8957E	0	–	0/3	0/3	0/9	0/9
Done Xangphai	Hauo Xangphai	14.0530N 105.8406E	105	–	2/3	0/3	7/9	0/9
	Hauo Xangphai	14.0513N 105.8444E	14	–	0/3	0/3	0/9	0/9
	Hauo Xangphai	14.0357N 105.8538E	91	–	2/3	1/3	6/9	3/9
	Hauo Xangphai	14.0352N 105.8540E	99	–	1/3	0/3	3/9	0/9
	Hauo Xangphai	14.0344N 105.8044E	88	–	3/3	1/3	9/9	2/9
Done Long	Haolong	14.0671N 105.7970E	63	–	1/3	0/3	4/9	0/9
	Longsong	14.0562N 105.8014E	71	–	3/3	1/3	9/9	3/9
	Longsong	14.0474N 105.8039E	20	-	2/3	0/3	6/9	0/9
	Longkang	14.0448N 105.8044E	21	–	3/3	2/3	9/9	6/9
	Longkang	14.0425N 105.8084E	90	–	2/3	1/3	6/9	3/9
Done Kadem	Kadem	14.0374N 105.7931E	1	–	1/3	0/3	3/9	0/9
	Kadem	14.0356N 105.7935E	0	–	2/3	2/3	6/9	6/9
	Kadem	14.0319N 105.7938E	57	–	3/3	0/3	9/9	0/9
	Kadem	14.0418N 105.7927E	111	–	2/3	0/3	6/9	0/9
	Kadem	14.0438N 105.7936E	59	–	2/3	0/3	6/9	0/9

For eDNA detection, three distinct water samples (biological replicates) were collected at each site. Each water sample was then analyzed using three independent qPCR reactions, resulting in nine technical replicates per site. Data are presented as the number of positive detections over the total number of replicates. “–” indicates that the survey was not conducted due to logistical unfeasibility caused by high water levels and rapid flow during the monsoon season

### Seasonal hydrophysical variations

**Fig. 3:**
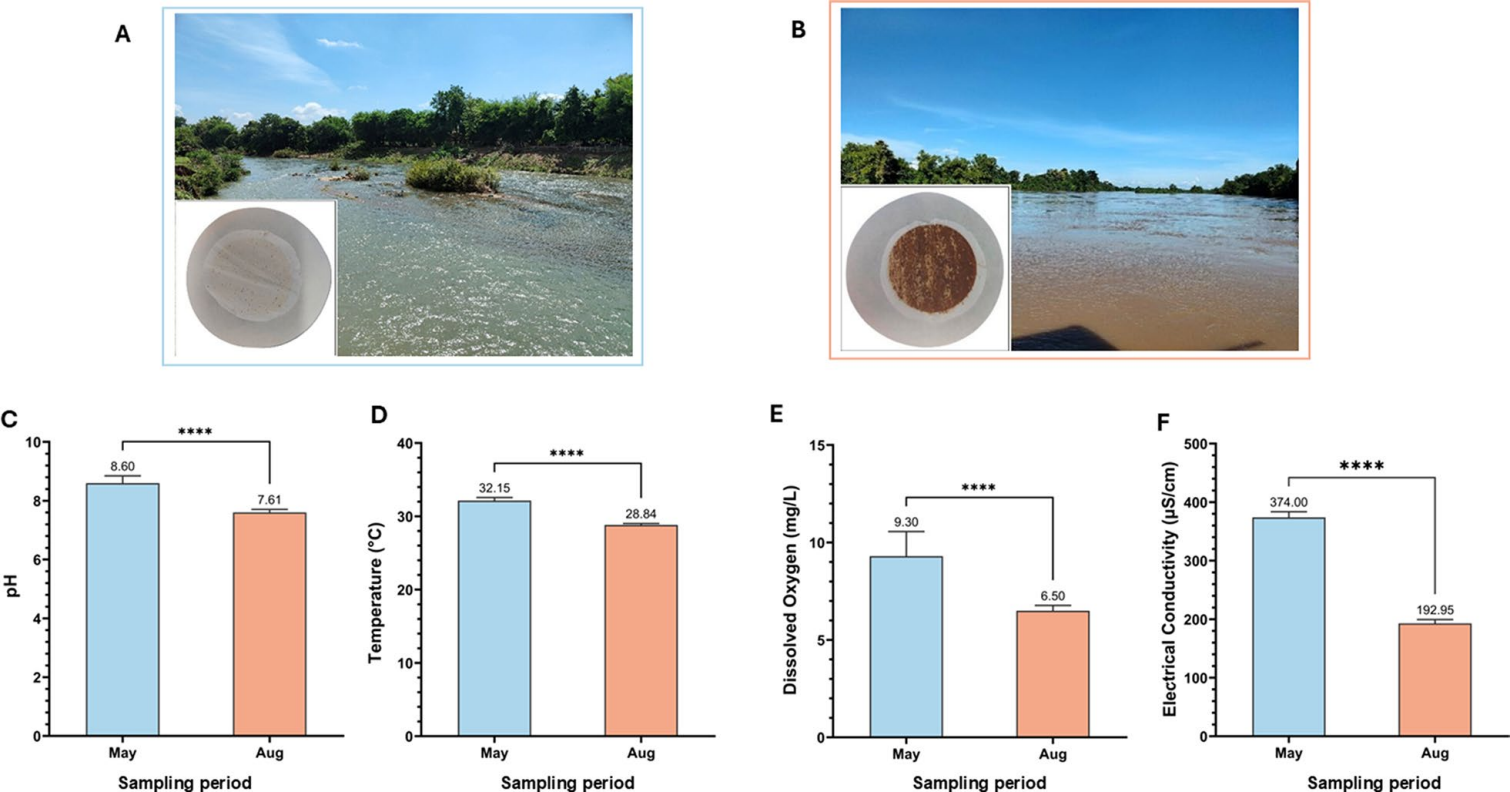
Seasonal variations in water characteristics of the Mekong River and membrane filtration performance. **A** Mekong River water during the dry season and associated membrane filtration performance; **B** Mekong River water during the wet season and associated membrane filtration performance; **C** Comparison of water pH levels between seasons; **D** Comparison of water temperature between seasons; **E** Comparison of water dissolved oxygen (DO) between seasons; **F** Comparison of water electrical conductivity between seasons. Blue bars represent dry season (May) measurements; orange bars represent wet season (August) measurements. Data are presented as Median and Interquartile Range

Quantitative analysis revealed significant differences between the dry (May) and wet (August) seasons across all measured parameters (Fig. 3; Mann-Whitney *U* test, all *P* < 0.05). When compared to the wet season, the dry season conditions exhibited: (i) higher pH (8.60 vs. 7.61); (ii) elevated temperature (32.15 °C vs. 28.84 °C); (iii) greater dissolved oxygen (9.30 mg/L vs. 6.50 mg/L); and (iv) higher electrical conductivity (374.00 μS/cm vs. 192.95 μS/cm). Hydrographic observations further indicated contrasting conditions between seasons: the dry season was characterized by clear, slow-flowing water, whereas the wet season featured turbid, fast-flowing water. These variations reflect distinct hydrological conditions influencing habitat suitability and the distribution of intermediate snail hosts.

### Environmental determinants of *N. aperta* distribution

**Fig. 4:**
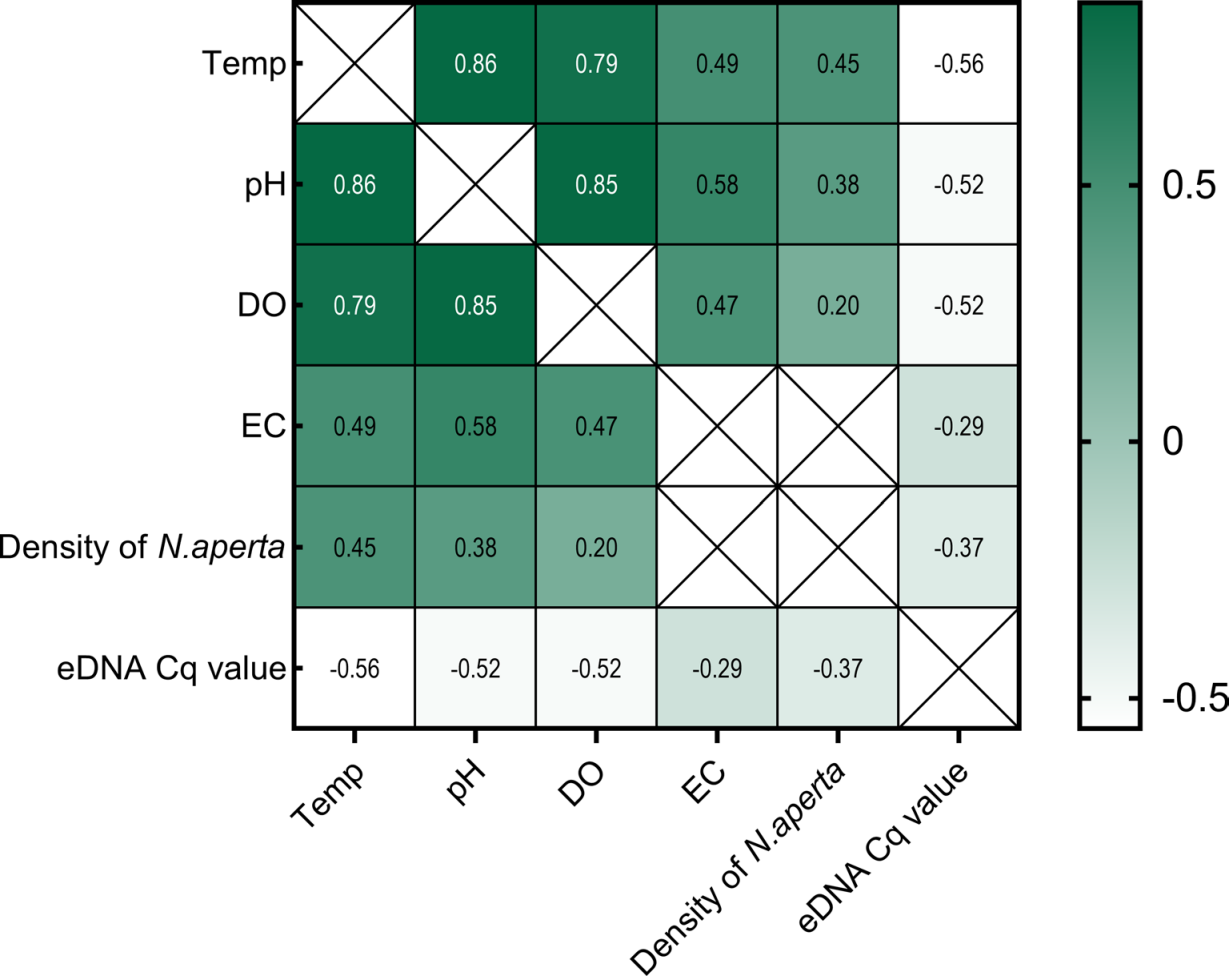
Spearman correlation matrix showing associations between *Neotricula aperta* density, eDNA distribution, and environmental determinants

Spearman’s rank correlation analysis revealed significant dry-season associations between *N. aperta* density and physicochemical parameters: water temperature (Spearman's *ρ* = 0.45; *P* < 0.01), pH (*ρ* = 0.38; *P* < 0.01), and dissolved oxygen (*ρ* = 0.20; *P* < 0.05). These findings suggest that *N. aperta* is more likely to colonize aquatic habitats with higher temperatures, elevated pH, and greater dissolved oxygen concentrations during the dry seasons. Moreover, a significant inverse relationship was also observed between Cq values and snail density (*ρ* = −0.37; *P* < 0.01), indicating that eDNA signal strength increases with greater snail abundance. Correspondingly, eDNA concentrations displayed a positive dose-dependent relationship with *N. aperta* density. In contrast, Cq values during the wet season were significantly higher than those in the dry season (*t* = − 9.66; *P* < 0.01), reflecting reduced eDNA detectability. This seasonal divergence was inversely correlated with all hydrological parameters (Fig. 4).

## Discussion

We developed a sensitive multiplex qPCR assay capable of detecting eDNA from *S. mekongi*-infected *N. aperta* within a 200 ml sub-sample under laboratory mesocosm conditions, addressing a critical gap in the environmental surveillance of *S. mekongi* transmission [32]. The concurrent detection of *S. mekongi* and *N. aperta* eDNA could provide molecular evidence of environmental transmission risk, whereas detection of *N. aperta* eDNA alone indicates snail presence and potential habitat suitability. In the present field survey, *S. mekongi* eDNA was not detected, consistent with the absence of infected snails in concurrent malacological surveys. Therefore, although this assay has potential for detecting *S. mekongi* in water bodies, its field performance for parasite detection requires further validation in sites with confirmed active transmission.

The selection of diagnostic targets for eDNA-based surveillance should prioritize genomic copy number to ensure maximum detection probability. Recent genomic characterization of *S. mekongi* revealed a highly repetitive genome with 806 rRNA copies, providing a substantially higher template concentration per cell [33]. While other potential mitochondrial targets, such as *cox1* and *nd5*, were also evaluated in our preliminary screening phase. For *N. aperta*, the selection of diagnostic markers is currently constrained by the limited availability of nuclear genomic resources in public databases. The 18S rRNA (*S. mekongi*) and 16S rRNA (*N. aperta*) loci were ultimately selected due to their consistently lower Cq values and superior analytical sensitivity. Through combined in silico and in situ validation, the primers and probes showed absolute specificity for *S. mekongi* and *N. aperta*. No cross-reactivity was observed with closely related or sympatric taxa, including other human-infecting *Schistosoma* species (specifically *S. haematobium*, *S. japonicum*, and *S. mansoni*) and a range of freshwater snails found in the Mekong Basin (detailed in Supplementary Table S2). Analytical sensitivity was standardized by calculating the LoD and LoQ following established eDNA methodologies [24]. When comparing with previous *Schistosoma* eDNA studies [21, 28, 34], we confirmed that our assay achieved sensitivity equal to or exceeding previously published methods (Supplementary Table S5). Therefore, our assay provides a potentially useful tool for surveillance in low-prevalence areas.

The sampling protocol for field water collection was established based on the physical characteristics of *S. mekongi* and *N. aperta* eDNA and the hydrological conditions of the Mekong River. Filtration remains the most widely adopted approach for capturing eDNA in aquatic systems, with membrane pore size being a key determinant of field performance[35]. Regarding the specific sampling volume, a recent review indicates that 500–1000 ml is the most common range employed in eDNA studies of neglected tropical diseases [36], thus, a volume of 1 L was selected for this study to ensure adequate detection sensitivity. Although smaller pore sizes can theoretically increase DNA capture efficiency, they are highly prone to clogging under field conditions with suspended sediments [37]. Increasing the number of filter changes would reduce clogging but at the cost of higher material use and increased labor during extraction and amplification. Studies have shown that filters with pore sizes greater than 3 μm are sufficient for schistosome eDNA capture in field settings [29], while 0.7 μm glass fiber filters have been widely used in schistosomiasis eDNA studies [17, 19]. Consequently, 0.7 μm filters were selected to achieve a balance between DNA capture efficiency and practical field feasibility. Controlled mesocosm experiments confirmed that 0.7 μm filters effectively captured extracellular snail eDNA, even under high-sediment conditions during the wet season, demonstrating strong field applicability. Furthermore, this study utilized standardized commercial sampling tools and kits, providing a reliable methodological reference for similar environmental DNA research.

Given that traditional malacological surveys are not considered as the gold standard for gastropod detection, we calculated concordance metrics to assess the field applicability of our eDNA approach. Our developed eDNA surveillance method showed moderate agreement with conventional snail sampling results, consistent with previous findings [19]. In our study, no *S. mekongi* eDNA was detected in field water samples. This result is consistent with the absence of *S. mekongi*-positive snails by crush-slide microscopy, although true absence cannot be confirmed because parasite eDNA detection may be affected by low cercarial output, dilution in the Mekong River, short cercarial lifespan, and PCR inhibition [38]. The detection of schistosomes in water depends on the presence of either whole cercariae or the transient DNA shed during their brief active life phase [39]. Given that cercariae have a short lifespan and the high dilution factor inherent in large lotic systems like the Mekong River [40], target DNA concentrations can rapidly diminish below detectable limits. While this molecular approach effectively overcomes a critical limitation of physical *N. aperta* sampling during monsoon-related high-water periods, certain limitations remain for direct parasite detection. The sensitivity of the assay can be influenced by environmental inhibitors and the large water volume of the Mekong Basin [41], which can lead to significant signal attenuation. These factors may result in false negatives when parasite density falls below the assay's detection threshold. Nevertheless, the assay’s ability to detect *N. aperta* eDNA across seasons suggests value for intermediate-host surveillance and may support future schistosomiasis monitoring programs.

Understanding hydrological influences on *N. aperta* distribution helps identify suitable snail habitats. However, the relatively uniform hydrological conditions (small standard deviations) across the Mekong River sampling sites during the same season limit precise modeling of these relationships. Previous studies have shown that low temperatures and high pH can slow eDNA degradation [27], while high sediment loads markedly reduce eDNA detection efficiency [42]. These factors likely explain the reduced number of eDNA-positive sites and higher Cq values observed during the wet season compared to the dry season. Our findings therefore highlight the importance of incorporating environmental variables when interpreting eDNA-based surveillance data. Although the relationship between biomass and eDNA concentration remains debated [43, 44], our data revealed a positive correlation between *N. aperta* density and eDNA concentration, suggesting that eDNA quantification may serve as a proxy for estimating snail abundance in natural habitats.

This study has several methodological constraints. First, the limited number of sampling sites and time points restricts generalization of the results. While the findings demonstrate the potential utility of eDNA technology for monitoring *N. aperta* distribution and possible *S. mekongi* transmission risk, the current evidence for field-based parasite detection remains exploratory. Broader spatial and temporal sampling will be needed to establish robust correlations between eDNA quantification, environmental parameters, and conventional snail data. Second, our multiplex qPCR assay cannot distinguish whether the *S. mekongi* DNA signal originates from shed eDNA or from whole cercariae. However, in the context of environmental surveillance, capturing both molecular and organismal DNA may actually enhance detection sensitivity, as it encompasses the total genetic load present in a given water body. Additional studies could employ size-fractionation techniques to characterize the distribution of these DNA sources, which would further refine the performance and interpretative power of the multiplex detection system. Third, we did not quantify eDNA degradation dynamics, a crucial knowledge gap for field applications. Although controlled studies suggest that eDNA may persist for up to one week [28], degradation rates in flowing systems inhabited by *N. aperta* may differ substantially. Future work should measure eDNA decay under natural hydrological conditions to refine sampling intervals. Finally, workflow optimization is essential for operational deployment. While commercial DNA extraction and qPCR kits enhance reproducibility, their performance for low-concentration eDNA in turbid tropical waters remains unverified. The Mekong River’s heavy sediment load may introduce PCR inhibitors not fully removed by current kits. Therefore, methodological refinements, particularly in eDNA capture and inhibitor removal, are necessary for routine application [45].

## Conclusions

This study developed a novel multiplex qPCR assay for the simultaneous detection of *S. mekongi* and its intermediate snail host *N. aperta.* Field evaluation in Lao PDR confirmed its utility for detecting *N. aperta* eDNA, while *S. mekongi* eDNA was not detected in field samples. Its capacity for reliable field detection of *S. mekongi* therefore requires further validation in settings with confirmed active transmission. As a laboratory-based molecular tool utilizing field-collected samples, this method offers a sensitive alternative to conventional surveys. Its integration into One Health surveillance could support WHO elimination targets, provided that necessary laboratory infrastructure and logistical support are available [46,47].

## Supplementary Information

Supplementary Material 1.

## Data Availability

The original contributions presented in the study are included in the article. Further inquiries can be directed to the corresponding author.

## Abbreviations

PCR: Polymerase chain reaction
DALYs: Disability-adjusted life years
WHO: World Health Organization
NTDs: Neglected tropical diseases
eDNA: Environmental DNA
LoD: Limit of detection
LoQ: Limit of quantification
NCBI: National Center for Biotechnology Information
BLAST: Basic local alignment search tool
MGB: Minor groove binder
