# Supplementary material for: Development and field evaluation of a multiplex qPCR assay for environmental DNA detection of Schistosoma mekongi and its intermediate snail host Neotricula aperta in the Mekong River Basin
Source: Infect Dis Poverty. 2026 Jun 4;15:65. doi: 10.1186/s40249-026-01466-1 (PMC13235101; doi:10.1186/s40249-026-01466-1)
Supplement: Supplementary file 1 — Supplementary Material 1. [file 40249_2026_1466_MOESM1_ESM.docx]

**Supplementary file**

Table S1. Primer-probe sets designed for the multiplex qPCR assay of *Neotricula aperta* and *Schistosoma mekongi*

| **Species** | **No.** | **Sequence (5'–3')** | **Fragment length** | **Reporter** | **Target gene** |
| --- | --- | --- | --- | --- | --- |
| *N. aperta* | 1 | F：AAACGGCCGCGGTACTCT  R：CCGTTCATACAAGCCCTCAATT  P：ACCGTGCAAAGGTAG | 74 | FAM | 16S rRNA |
|  | 2 | F：GGCTGGTACAGGATGAACAGTTT  R：TCCACCTGCATGGGCTAAA  P：TCCTCCACTTGCTGGAA | 61 |  | COX1 |
|  | 3 | F：GGGCTGGTACAGGATGAACAG  R：AGACCCTCCTGCATGAGCTAAG  P：TTATCCTCCACTTGCTGGAA | 65 |  | COX1 |
|  | 4 | F：TAAACGGCCGCGGTACTCT  R：CCGTTCATACAAGCCCTCAATT  P：ACCGTGCAAAGGTAG | 75 |  | 16S rRNA |
| *S. mekongi* | 5 | F：CATGCACCTGGCCTTGTG  R：TCGCTGCAGCCTAGGATATTTAC  P：TGCATGTACGCTGGCT | 68 | VIC | 18S rRNA |
|  | 6 | F：GGTTCCGGTGTTGGTTGAAC  R：ATAATCTACACCTACACCAGACGTAGCT  P：TTTATCCTCCGTTGTCTTC | 72 |  | COX1 |
|  | 7 | F：TGTGGTGGATATGCGAATTCA  R：GGTGTAGGAGCACGCATAGCT  P：TTGTTTCTACGAAGAGTGC | 119 |  | ND5 |
|  | 8 | F：TGGTTCCGGTGTTGGTTGA  R：CACCTACACCAGACGTAGCTTGA  P：CTTTTTATCCTCCGTTGTCTT | 65 |  | COX1 |

F: Forward primer; R: Reverse primer; P: Probe.

Table S2. Common mollusc species in the Mekong River Basin

| **No.** | **Picture** | **Name** |
| --- | --- | --- |
| 1 | 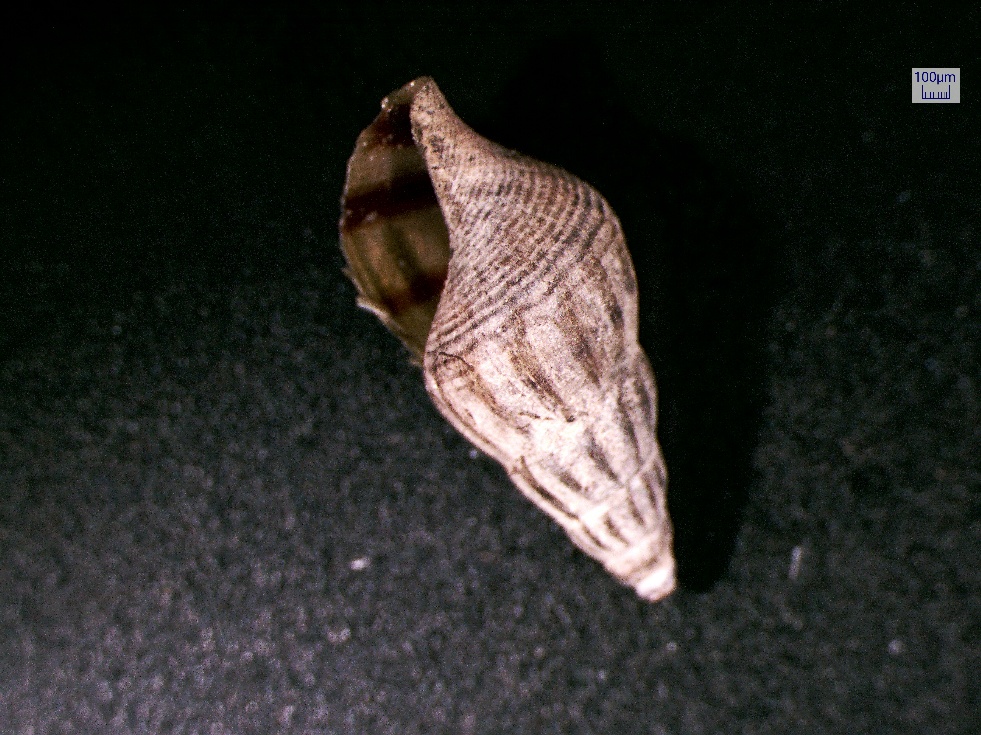 | *Anentome sp.* |
| 2 | 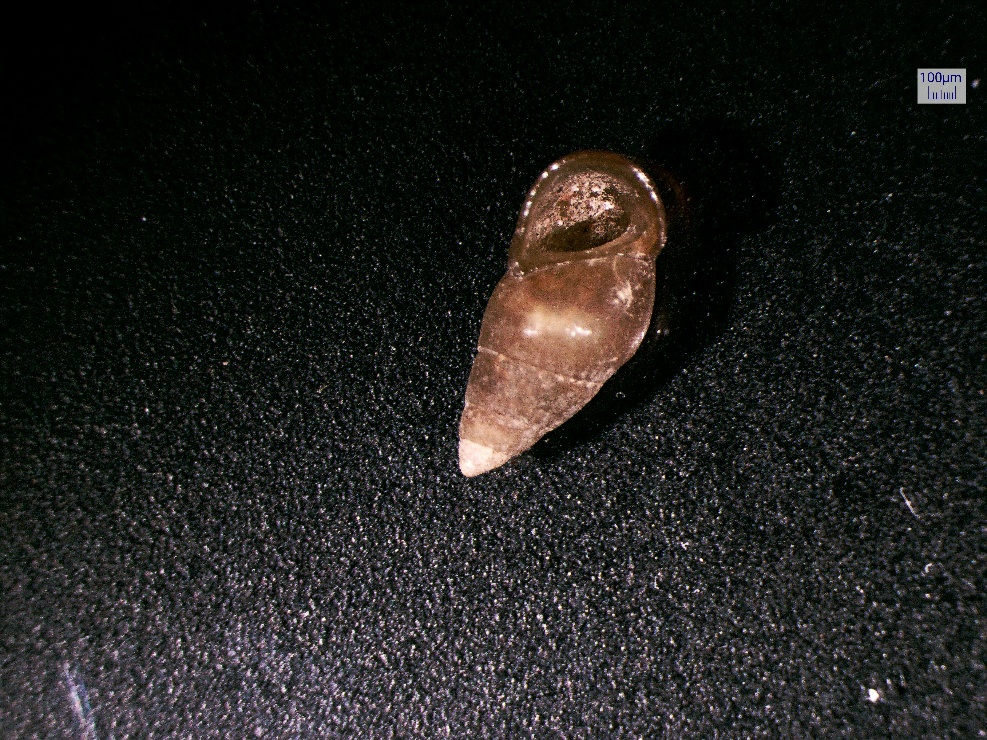 | *Bithynia sp.* |
| 3 | 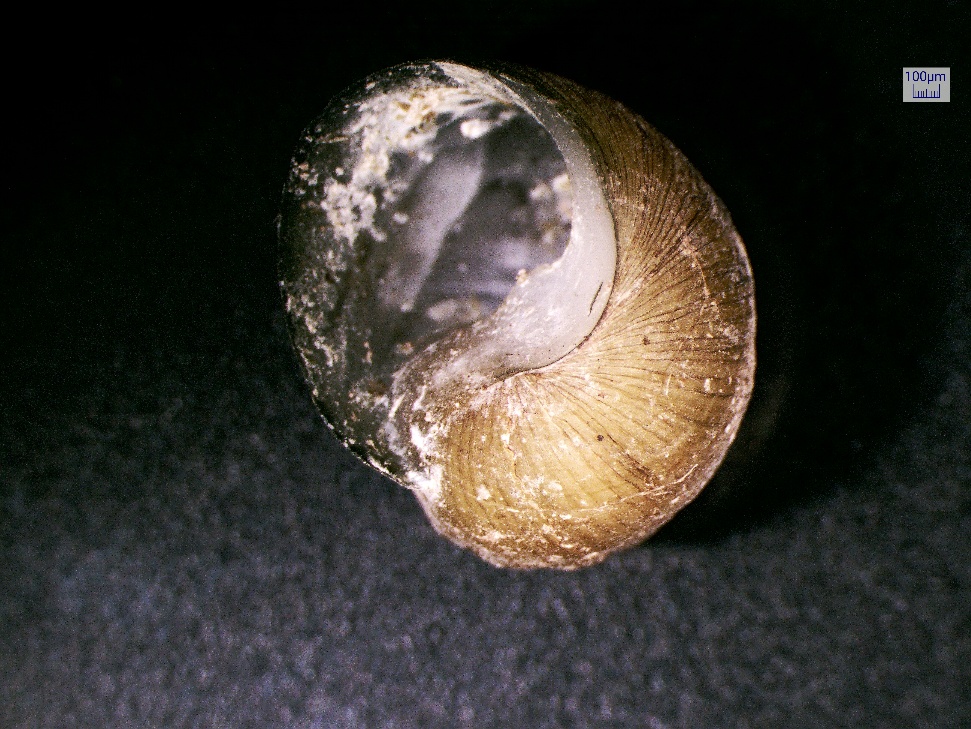 | *Vittina sp.* |
| 4 | 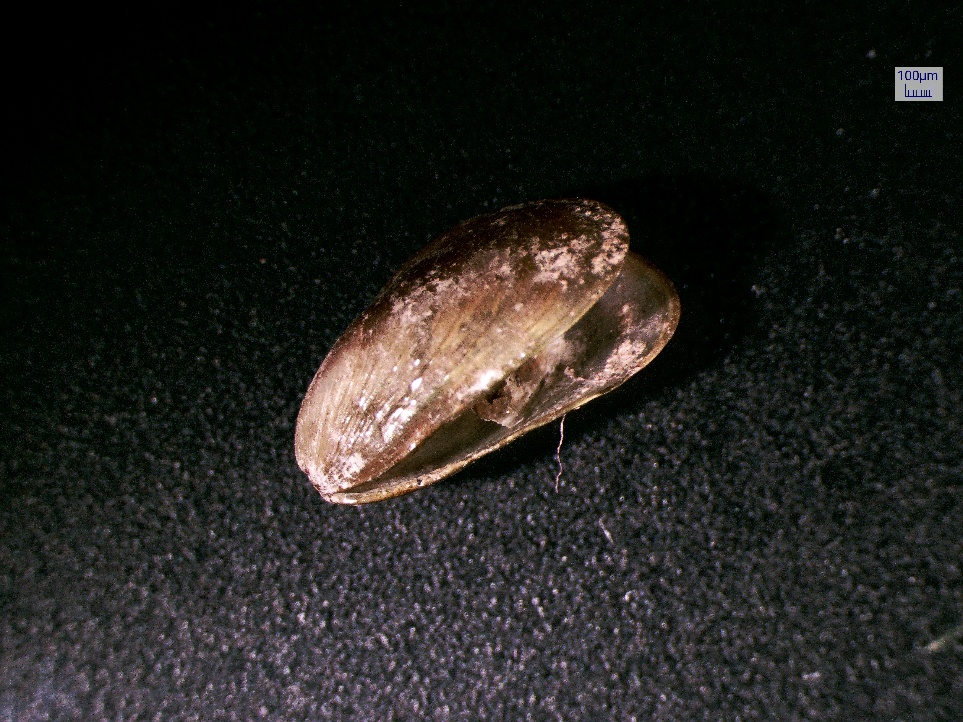 | *Bineurus sp*. |
| 5 | 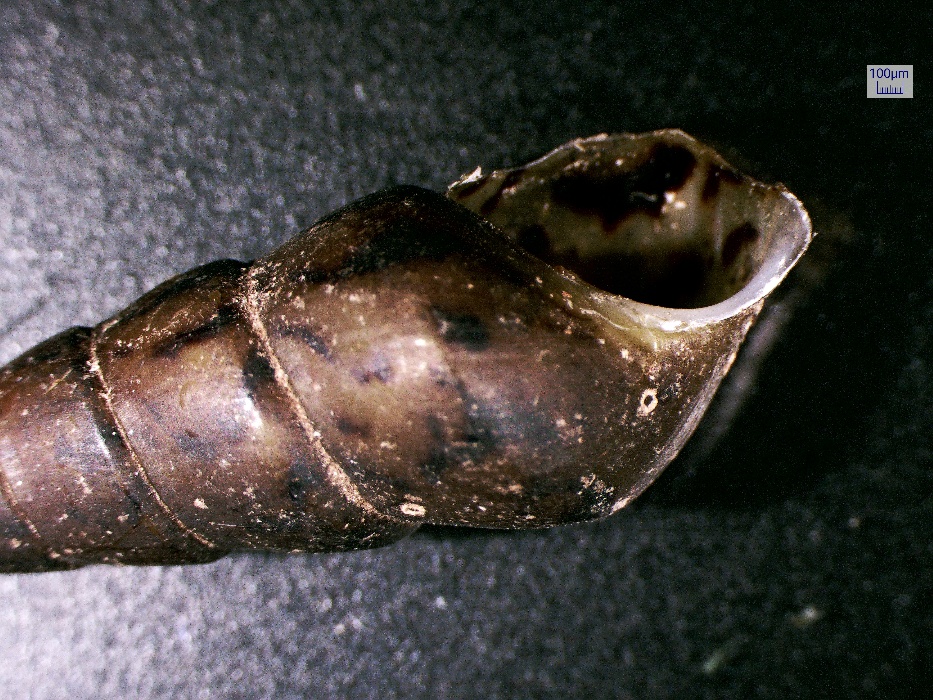 | *Sulcospira sp.* |
| 6 | 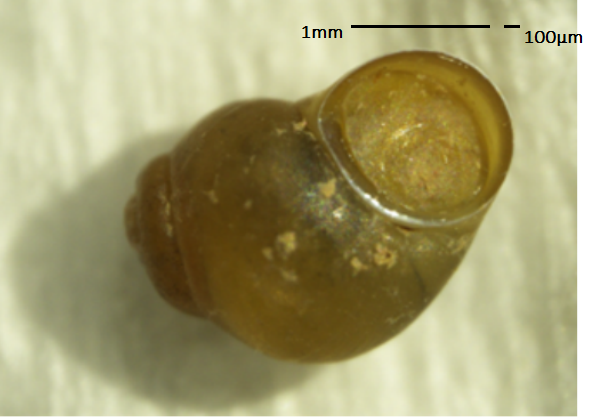 | *Stenothyra sp.* |
| 7 | 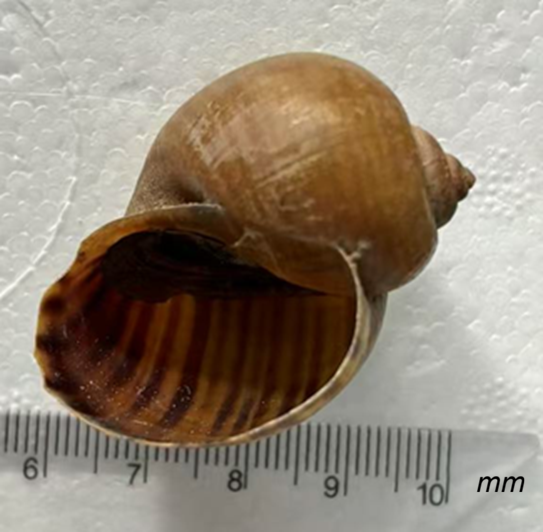 | *Pomacea sp.* |
| 8 | 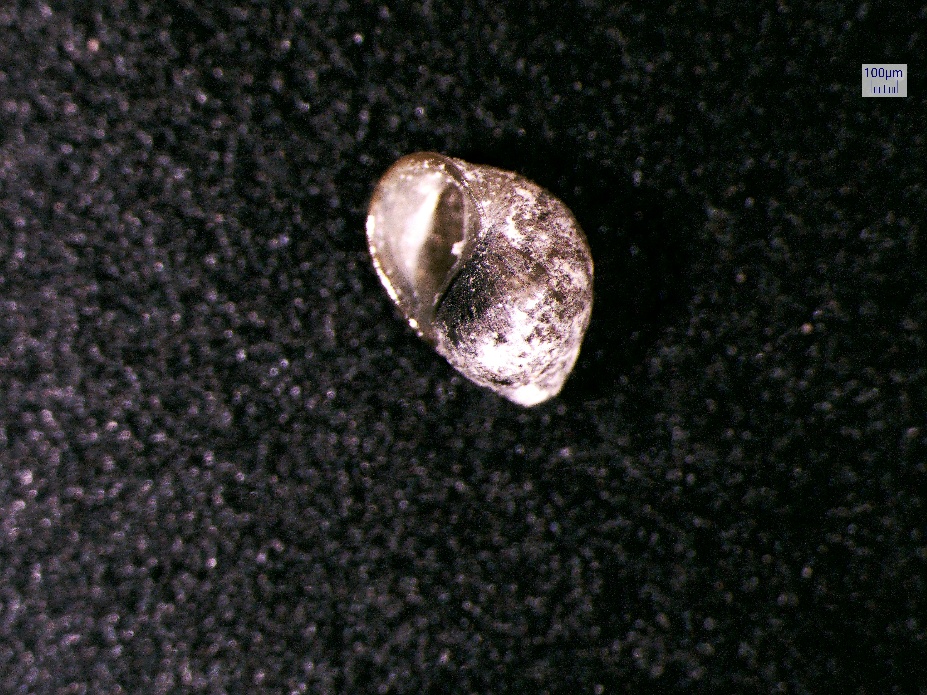 | *Neotricula aperta* |

Table S3. Comparison of Cq values between singleplex and multiplex qPCR assays using genomic DNA from infected *N. aperta*

| **qPCR type** | **Primer-probe sets No.** | **Mean Cq value** | | |  | **ΔCq Shift** | | |
| --- | --- | --- | --- | --- | --- | --- | --- | --- |
|  |  | **FAM, *N. aperta*** | **VIC, *S. mekongi*** |  | | **FAM, *N. aperta*** | **FAM, *N. aperta*** |  |
| Singleplex | 1 | 12.58 | - |  | |  |  |  |
|  | 2 | 17.35 | - |  | |  |  |  |
|  | 3 | 20.01 | - |  | |  |  |  |
|  | 4 | 14.32 | - |  | |  |  |  |
|  | 5 | - | 20.48 |  | |  |  |  |
|  | 6 | - | 23.63 |  | |  |  |  |
|  | 7 | - | 24.03 |  | |  |  |  |
|  | 8 | - | 19.91 |  | |  |  |  |
| Multiplex | **1+5** | **13.03** | **20.92** |  | | **0.45** | **0.44** |  |
|  | 1+6 | 12.93 | 25.54 |  | | 0.35 | 1.91 |  |
|  | 1+7 | 25.18 | 26.11 |  | | 12.60 | 2.08 |  |
|  | 1+8 | 13.41 | 28.22 |  | | 0.83 | 8.31 |  |
|  | 2+5 | 19.09 | 21.73 |  | | 1.74 | 1.25 |  |
|  | 2+6 | 21.71 | 26.02 |  | | 4.36 | 2.39 |  |
|  | 2+7 | 20.88 | 28.35 |  | | 3.53 | 4.32 |  |
|  | 2+8 | 20.23 | 27.11 |  | | 2.88 | 7.20 |  |
|  | 3+5 | 26.35 | 23.24 |  | | 6.34 | 2.76 |  |
|  | 3+6 | 24.75 | 29.26 |  | | 4.74 | 5.63 |  |
|  | 3+7 | 24.79 | 28.34 |  | | 4.78 | 4.31 |  |
|  | 3+8 | 25.33 | 29.68 |  | | 5.32 | 9.77 |  |
|  | 4+5 | 15.05 | 23.85 |  | | 0.73 | 3.37 |  |
|  | 4+6 | 14.70 | 28.00 |  | | 0.38 | 4.37 |  |
|  | 4+7 | 15.38 | 27.30 |  | | 1.06 | 3.27 |  |
|  | 4+8 | 14.75 | 29.60 |  | | 0.43 | 9.69 |  |

"–", no amplification; The bold row represents the optimal multiplex primer-probe combination determined for further validation and field applications.

| *Schistosoma mekongi*: |
| --- |
| 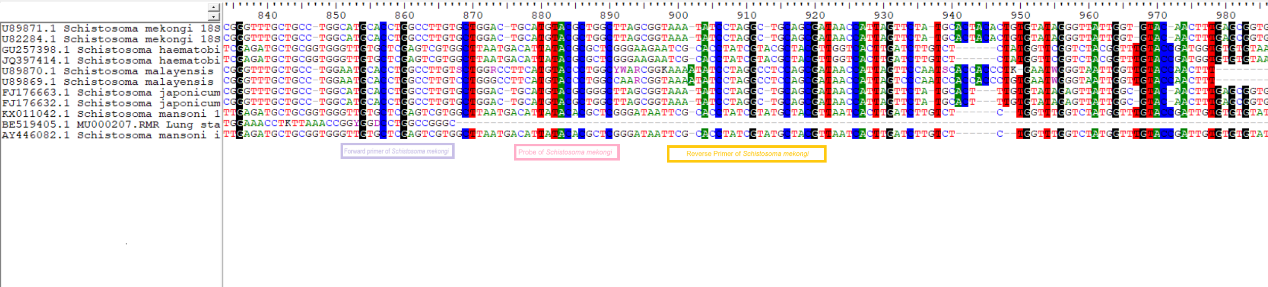 |
| *Neotricula aperta*: |
| 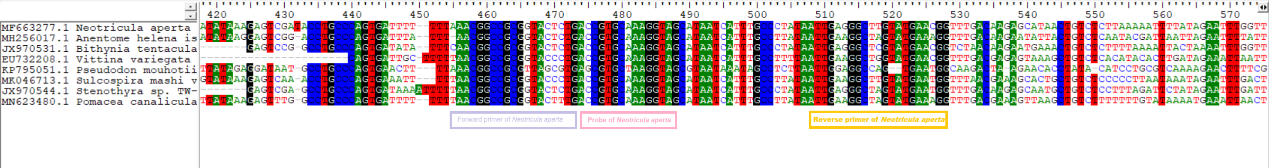 |

Figure S1. In silico specificity assessment of the optimized primer-probe sets for *S. mekongi* (No.5, table S1) and *N. aperta* (No.1, table S1) against non-target species.


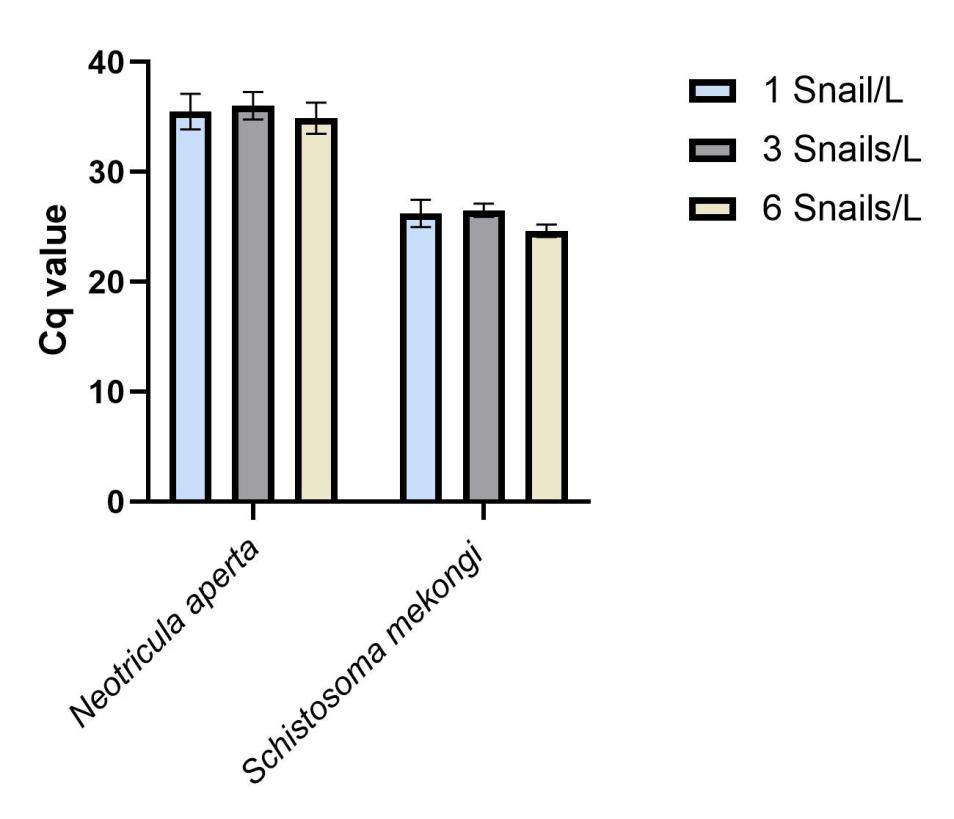


Figure S2. Multiplex qPCR performance in controlled aquaria with infected *N. aperta.*

Table S4. Cq value of standard curve

| Well | Fluor | Sample | Cq | SQ | Target |
| --- | --- | --- | --- | --- | --- |
| A07 | VIC | NTC | - | - | *Schistosoma mekongi* |
| A08 | VIC | NTC | - | - | *Schistosoma mekongi* |
| A09 | VIC | NTC | - | - | *Schistosoma mekongi* |
| A10 | VIC | NTC | - | - | *Schistosoma mekongi* |
| A11 | VIC | NTC | - | - | *Schistosoma mekongi* |
| A12 | VIC | NTC | - | - | *Schistosoma mekongi* |
| A07 | VIC | NTC | - | - | *Schistosoma mekongi* |
| A08 | VIC | NTC | - | - | *Schistosoma mekongi* |
| A09 | VIC | NTC | - | - | *Schistosoma mekongi* |
| A10 | VIC | NTC | - | - | *Schistosoma mekongi* |
| A11 | VIC | NTC | - | - | *Schistosoma mekongi* |
| A12 | VIC | NTC | - | - | *Schistosoma mekongi* |
| A07 | VIC | NTC | - | - | *Schistosoma mekongi* |
| A08 | VIC | NTC | - | - | *Schistosoma mekongi* |
| A09 | VIC | NTC | - | - | *Schistosoma mekongi* |
| A10 | VIC | NTC | - | - | *Schistosoma mekongi* |
| A11 | VIC | NTC | - | - | *Schistosoma mekongi* |
| A12 | VIC | NTC | - | - | *Schistosoma mekongi* |
| A01 | FAM | NTC | - | - | *Neotricula aperta* |
| A02 | FAM | NTC | - | - | *Neotricula aperta* |
| A03 | FAM | NTC | - | - | *Neotricula aperta* |
| A04 | FAM | NTC | - | - | *Neotricula aperta* |
| A05 | FAM | NTC | - | - | *Neotricula aperta* |
| A06 | FAM | NTC | - | - | *Neotricula aperta* |
| A01 | FAM | NTC | - | - | *Neotricula aperta* |
| A02 | FAM | NTC | - | - | *Neotricula aperta* |
| A03 | FAM | NTC | - | - | *Neotricula aperta* |
| A04 | FAM | NTC | - | - | *Neotricula aperta* |
| A05 | FAM | NTC | - | - | *Neotricula aperta* |
| A06 | FAM | NTC | - | - | *Neotricula aperta* |
| A01 | FAM | NTC | - | - | *Neotricula aperta* |
| A02 | FAM | NTC | - | - | *Neotricula aperta* |
| A03 | FAM | NTC | - | - | *Neotricula aperta* |
| A04 | FAM | NTC | - | - | *Neotricula aperta* |
| A05 | FAM | NTC | - | - | *Neotricula aperta* |
| A06 | FAM | NTC | - | - | *Neotricula aperta* |
| F05 | VIC | STD_1000000 | 18.12 | 1000000 | *Schistosoma mekongi* |
| G05 | VIC | STD_1000000 | 18.01 | 1000000 | *Schistosoma mekongi* |
| H05 | VIC | STD_1000000 | 17.68 | 1000000 | *Schistosoma mekongi* |
| F05 | VIC | STD_1000000 | 17.86 | 1000000 | *Schistosoma mekongi* |
| G05 | VIC | STD_1000000 | 18.31 | 1000000 | *Schistosoma mekongi* |
| H05 | VIC | STD_1000000 | 18.41 | 1000000 | *Schistosoma mekongi* |
| F05 | VIC | STD_1000000 | 18.67 | 1000000 | *Schistosoma mekongi* |
| G05 | VIC | STD_1000000 | 18.03 | 1000000 | *Schistosoma mekongi* |
| H05 | VIC | STD_1000000 | 17.72 | 1000000 | *Schistosoma mekongi* |
| C05 | FAM | STD_1000000 | 18.57 | 1000000 | *Neotricula aperta* |
| D05 | FAM | STD_1000000 | 19.00 | 1000000 | *Neotricula aperta* |
| E05 | FAM | STD_1000000 | 18.79 | 1000000 | *Neotricula aperta* |
| C05 | FAM | STD_1000000 | 18.86 | 1000000 | *Neotricula aperta* |
| D05 | FAM | STD_1000000 | 18.24 | 1000000 | *Neotricula aperta* |
| E05 | FAM | STD_1000000 | 17.97 | 1000000 | *Neotricula aperta* |
| C05 | FAM | STD_1000000 | 19.63 | 1000000 | *Neotricula aperta* |
| D05 | FAM | STD_1000000 | 19.44 | 1000000 | *Neotricula aperta* |
| E05 | FAM | STD_1000000 | 19.56 | 1000000 | *Neotricula aperta* |
| F06 | VIC | STD_100000 | 21.53 | 100000 | *Schistosoma mekongi* |
| G06 | VIC | STD_100000 | 21.31 | 100000 | *Schistosoma mekongi* |
| H06 | VIC | STD_100000 | 20.93 | 100000 | *Schistosoma mekongi* |
| F06 | VIC | STD_100000 | 21.56 | 100000 | *Schistosoma mekongi* |
| G06 | VIC | STD_100000 | 21.56 | 100000 | *Schistosoma mekongi* |
| H06 | VIC | STD_100000 | 20.86 | 100000 | *Schistosoma mekongi* |
| F06 | VIC | STD_100000 | 22.03 | 100000 | *Schistosoma mekongi* |
| G06 | VIC | STD_100000 | 21.70 | 100000 | *Schistosoma mekongi* |
| H06 | VIC | STD_100000 | 21.36 | 100000 | *Schistosoma mekongi* |
| C06 | FAM | STD_100000 | 22.83 | 100000 | *Neotricula aperta* |
| D06 | FAM | STD_100000 | 22.66 | 100000 | *Neotricula aperta* |
| E06 | FAM | STD_100000 | 22.26 | 100000 | *Neotricula aperta* |
| C06 | FAM | STD_100000 | 22.44 | 100000 | *Neotricula aperta* |
| D06 | FAM | STD_100000 | 21.97 | 100000 | *Neotricula aperta* |
| E06 | FAM | STD_100000 | 22.39 | 100000 | *Neotricula aperta* |
| C06 | FAM | STD_100000 | 22.84 | 100000 | *Neotricula aperta* |
| D06 | FAM | STD_100000 | 22.97 | 100000 | *Neotricula aperta* |
| E06 | FAM | STD_100000 | 22.68 | 100000 | *Neotricula aperta* |
| F07 | VIC | STD_10000 | 25.12 | 10000 | *Schistosoma mekongi* |
| G07 | VIC | STD_10000 | 24.79 | 10000 | *Schistosoma mekongi* |
| H07 | VIC | STD_10000 | 24.42 | 10000 | *Schistosoma mekongi* |
| F07 | VIC | STD_10000 | 24.82 | 10000 | *Schistosoma mekongi* |
| G07 | VIC | STD_10000 | 25.32 | 10000 | *Schistosoma mekongi* |
| H07 | VIC | STD_10000 | 25.02 | 10000 | *Schistosoma mekongi* |
| F07 | VIC | STD_10000 | 24.72 | 10000 | *Schistosoma mekongi* |
| G07 | VIC | STD_10000 | 24.27 | 10000 | *Schistosoma mekongi* |
| H07 | VIC | STD_10000 | 25.03 | 10000 | *Schistosoma mekongi* |
| C07 | FAM | STD_10000 | 25.53 | 10000 | *Neotricula aperta* |
| D07 | FAM | STD_10000 | 25.56 | 10000 | *Neotricula aperta* |
| E07 | FAM | STD_10000 | 25.71 | 10000 | *Neotricula aperta* |
| C07 | FAM | STD_10000 | 25.29 | 10000 | *Neotricula aperta* |
| D07 | FAM | STD_10000 | 25.81 | 10000 | *Neotricula aperta* |
| E07 | FAM | STD_10000 | 25.67 | 10000 | *Neotricula aperta* |
| C07 | FAM | STD_10000 | 25.26 | 10000 | *Neotricula aperta* |
| D07 | FAM | STD_10000 | 25.31 | 10000 | *Neotricula aperta* |
| E07 | FAM | STD_10000 | 25.39 | 10000 | *Neotricula aperta* |
| F08 | VIC | STD_1000 | 27.96 | 1000 | *Schistosoma mekongi* |
| G08 | VIC | STD_1000 | 28.47 | 1000 | *Schistosoma mekongi* |
| H08 | VIC | STD_1000 | 28.51 | 1000 | *Schistosoma mekongi* |
| F08 | VIC | STD_1000 | 28.11 | 1000 | *Schistosoma mekongi* |
| G08 | VIC | STD_1000 | 28.39 | 1000 | *Schistosoma mekongi* |
| H08 | VIC | STD_1000 | 27.72 | 1000 | *Schistosoma mekongi* |
| F08 | VIC | STD_1000 | 28.04 | 1000 | *Schistosoma mekongi* |
| G08 | VIC | STD_1000 | 27.93 | 1000 | *Schistosoma mekongi* |
| H08 | VIC | STD_1000 | 27.85 | 1000 | *Schistosoma mekongi* |
| C08 | FAM | STD_1000 | 29.11 | 1000 | *Neotricula aperta* |
| D08 | FAM | STD_1000 | 28.62 | 1000 | *Neotricula aperta* |
| E08 | FAM | STD_1000 | 28.40 | 1000 | *Neotricula aperta* |
| C08 | FAM | STD_1000 | 28.96 | 1000 | *Neotricula aperta* |
| D08 | FAM | STD_1000 | 29.10 | 1000 | *Neotricula aperta* |
| E08 | FAM | STD_1000 | 29.05 | 1000 | *Neotricula aperta* |
| C08 | FAM | STD_1000 | 29.46 | 1000 | *Neotricula aperta* |
| D08 | FAM | STD_1000 | 29.32 | 1000 | *Neotricula aperta* |
| E08 | FAM | STD_1000 | 29.15 | 1000 | *Neotricula aperta* |
| F09 | VIC | STD_100 | 31.87 | 100 | *Schistosoma mekongi* |
| G09 | VIC | STD_100 | 32.10 | 100 | *Schistosoma mekongi* |
| H09 | VIC | STD_100 | 31.67 | 100 | *Schistosoma mekongi* |
| F09 | VIC | STD_100 | 32.03 | 100 | *Schistosoma mekongi* |
| G09 | VIC | STD_100 | 31.68 | 100 | *Schistosoma mekongi* |
| H09 | VIC | STD_100 | 31.20 | 100 | *Schistosoma mekongi* |
| F09 | VIC | STD_100 | 31.69 | 100 | *Schistosoma mekongi* |
| G09 | VIC | STD_100 | 32.29 | 100 | *Schistosoma mekongi* |
| H09 | VIC | STD_100 | 31.22 | 100 | *Schistosoma mekongi* |
| C09 | FAM | STD_100 | 31.98 | 100 | *Neotricula aperta* |
| D09 | FAM | STD_100 | 32.53 | 100 | *Neotricula aperta* |
| E09 | FAM | STD_100 | 32.37 | 100 | *Neotricula aperta* |
| C09 | FAM | STD_100 | 32.48 | 100 | *Neotricula aperta* |
| D09 | FAM | STD_100 | 32.54 | 100 | *Neotricula aperta* |
| E09 | FAM | STD_100 | 32.22 | 100 | *Neotricula aperta* |
| C09 | FAM | STD_100 | 32.25 | 100 | *Neotricula aperta* |
| D09 | FAM | STD_100 | 32.05 | 100 | *Neotricula aperta* |
| E09 | FAM | STD_100 | 32.43 | 100 | *Neotricula aperta* |
| F10 | VIC | STD_10 | 34.57 | 10 | *Schistosoma mekongi* |
| G10 | VIC | STD_10 | 35.17 | 10 | *Schistosoma mekongi* |
| H10 | VIC | STD_10 | 35.21 | 10 | *Schistosoma mekongi* |
| F10 | VIC | STD_10 | 33.94 | 10 | *Schistosoma mekongi* |
| G10 | VIC | STD_10 | 34.87 | 10 | *Schistosoma mekongi* |
| H10 | VIC | STD_10 | 34.97 | 10 | *Schistosoma mekongi* |
| F10 | VIC | STD_10 | 35.25 | 10 | *Schistosoma mekongi* |
| G10 | VIC | STD_10 | 36.08 | 10 | *Schistosoma mekongi* |
| H10 | VIC | STD_10 | 34.34 | 10 | *Schistosoma mekongi* |
| C10 | FAM | STD_10 | 35.75 | 10 | *Neotricula aperta* |
| D10 | FAM | STD_10 | 34.73 | 10 | *Neotricula aperta* |
| E10 | FAM | STD_10 | 35.85 | 10 | *Neotricula aperta* |
| C10 | FAM | STD_10 | 34.50 | 10 | *Neotricula aperta* |
| D10 | FAM | STD_10 | 35.37 | 10 | *Neotricula aperta* |
| E10 | FAM | STD_10 | 36.49 | 10 | *Neotricula aperta* |
| C10 | FAM | STD_10 | 33.75 | 10 | *Neotricula aperta* |
| D10 | FAM | STD_10 | 35.98 | 10 | *Neotricula aperta* |
| E10 | FAM | STD_10 | 35.73 | 10 | *Neotricula aperta* |
| F11 | VIC | STD_1 | - | 1 | *Schistosoma mekongi* |
| G11 | VIC | STD_1 | - | 1 | *Schistosoma mekongi* |
| H11 | VIC | STD_1 | - | 1 | *Schistosoma mekongi* |
| F11 | VIC | STD_1 | - | 1 | *Schistosoma mekongi* |
| G11 | VIC | STD_1 | - | 1 | *Schistosoma mekongi* |
| H11 | VIC | STD_1 | - | 1 | *Schistosoma mekongi* |
| F11 | VIC | STD_1 | - | 1 | *Schistosoma mekongi* |
| G11 | VIC | STD_1 | 37.70 | 1 | *Schistosoma mekongi* |
| H11 | VIC | STD_1 | 37.70 | 1 | *Schistosoma mekongi* |
| C11 | FAM | STD_1 | 38.70 | 1 | *Neotricula aperta* |
| D11 | FAM | STD_1 | - | 1 | *Neotricula aperta* |
| E11 | FAM | STD_1 | 39.16 | 1 | *Neotricula aperta* |
| C11 | FAM | STD_1 | - | 1 | *Neotricula aperta* |
| D11 | FAM | STD_1 | - | 1 | *Neotricula aperta* |
| E11 | FAM | STD_1 | 39.15 | 1 | *Neotricula aperta* |
| C11 | FAM | STD_1 | - | 1 | *Neotricula aperta* |
| D11 | FAM | STD_1 | 39.08 | 1 | *Neotricula aperta* |
| E11 | FAM | STD_1 | - | 1 | *Neotricula aperta* |

"–", no amplification.

Table S5. Detection ability in similar eDNA studies of schistosomiasis

| No. | Title | Target species | Test technology | Limit of detection | Limit of quantification |
| --- | --- | --- | --- | --- | --- |
| 1[1] | Environmental DNA-based xenomonitoring for determining *Schistosoma* presence in tropical freshwaters | *Schistosoma mansoni* and *S. haematobium* | qPCR | *S. mansoni*: 32.36 copies/μl;  *S. haematobium*: 1.33 copies/μl | *S. mansoni*: 100 copies/μl;  *S. haematobium*: 100 copies/μl |
| 2[2] | Environmental DNA for improved detection and environmental surveillance of schistosomiasis | *S. mansoni* | qPCR | 1 copy/μl level | 10 copies/μl level |
| 3[3] | Development of environmental loop-mediated isothermal amplification (eLAMP) diagnostic tool for Bulinus truncatus field detection | *Bulinus truncatus* | LAMP | 70 copies/μl; | - |

**Reference:**

1. Alzaylaee, H.; Collins, R. A.; Shechonge, A.; Ngatunga, B. P.; Morgan, E. R.; Genner, M. J., Environmental DNA-based xenomonitoring for determining Schistosoma presence in tropical freshwaters. *Parasites & vectors* **2020,** *13*, (1), 63.

2. Sengupta, M. E.; Hellström, M.; Kariuki, H. C.; Olsen, A.; Thomsen, P. F.; Mejer, H.; Willerslev, E.; Mwanje, M. T.; Madsen, H.; Kristensen, T. K., Environmental DNA for improved detection and environmental surveillance of schistosomiasis. *Proceedings of the National Academy of Sciences* **2019,** *116*, (18), 8931-8940.

3. Blin, M.; Senghor, B.; Boissier, J.; Mulero, S.; Rey, O.; Portela, J., Development of environmental loop-mediated isothermal amplification (eLAMP) diagnostic tool for Bulinus truncatus field detection. *Parasites & Vectors* **2023,** *16*, (1), 78.
